# The ability of algal organic matter and surface runoff to promote the abundance of pathogenic and non-pathogenic strains of *Vibrio parahaemolyticus* in Long Island Sound, USA

**DOI:** 10.1371/journal.pone.0185994

**Published:** 2017-10-11

**Authors:** Jake D. Thickman, Christopher J. Gobler

**Affiliations:** School of Marine and Atmospheric Sciences at Stony Brook University, Southampton, New York, United States of America; Universidade de Aveiro, PORTUGAL

## Abstract

Food safety is a major concern in the shellfish industry, as severe illness can result from consuming shellfish that have accumulated waterborne pathogens. Shellfish harvesting areas are typically monitored for indicator bacteria such as fecal coliforms that serve as proxies for enteric pathogens although these indicators have shown little relation to some naturally occurring pathogenic bacteria such as *Vibrio parahaemolyticus*. To examine the dynamics and ecology of pathogenic and non-pathogenic strains of *V*. *parahaemolyticus* and address the relevance of indicator bacteria in predicting *V*. *parahaemolyticus* concentrations, field surveys and experiments were carried out in western Long Island Sound, NY, USA, a region that has experienced recent outbreaks of shellfish contaminated with *V*. *parahaemolyticus*. Pathogenic and non-pathogenic strains were quantified via PCR detection of marker genes and most probable number techniques. Field survey data showed little correspondence between fecal coliforms and *V*. *parahaemolyticus*, but significant correlations between *V*. *parahaemolyticus* and an alternative indicator, enterococci, and between *V*. *parahaemolyticus* and short-term (48 h) rainfall were observed. Experiments demonstrated that enrichment of seawater with phytoplankton-derived dissolved organic matter significantly increased the concentration of total *V*. *parahaemolyticus* and the presence pathogenic *V*. *parahaemolyticus*, but higher temperatures did not. Collectively, these study results suggest that fecal coliforms may fail to account for the full suite of important shellfish pathogens but that enterococci could provide a potential alternative or supplement to shellfish sanitation monitoring. Given the ability of algal-derived dissolved organic matter to promote the growth of pathogenic *V*. *parahaemolyticus*, restricting nutrient inputs into coastal water bodies that promote algal blooms may indirectly decrease the proliferation of *V*. *parahaemolyticus* and protect public health.

## Introduction

Shellfish harvesting is a billion-dollar industry in the US. Due to the health risks, including gastroenteritis and in extreme cases mortality, associated with consuming shellfish containing pathogenic microbes, food safety is a high priority in the industry [1–3]. To ensure the safety of shellfish for human consumption, water quality standards are used to designate areas of coastal zones that are fit for shellfish harvest based on potential shellfish exposure to pathogenic microbes. When filtering particles from water bodies, bivalves may accumulate microbial pathogens present into their tissues at concentrations many times greater than ambient levels [3–4] that originate from wastewater contamination or natural sources [5–6]. Due to the complexity, time, and expense of identifying and measuring multiple pathogenic microbes in coastal waters [7], fecal coliform bacteria are typically used as indicator organisms in water quality surveys. If fecal coliform bacteria are found at high concentrations, it is assumed that enteric pathogens that can accumulate in shellfish tissues and sicken consumers will also be present and thus may have contaminated local shellfish stocks [8–9]. To function as effective indicator organisms, fecal coliform bacteria should not reproduce in contaminated environments and should also be correlated with the presence of pathogens [9].

While fecal coliform bacteria standards for shellfish harvest are common, such a metric does not necessarily capture the full spectrum of pathogenic microbes that can accumulate in shellfish tissues [9], and it may be a particularly poor proxy for *Vibrio parahaemolyticus*, which occurs naturally in estuaries and has been identified as a pathogen of concern in recent years in coastal waters [10–12]. *V*. *parahaemolyticus* is a gram-negative, curved, rod shaped bacterium. The species is halophilic and broadly distributed, inhabiting marine and estuarine waters in the Atlantic [13–15], Pacific [16], and Indian Oceans [17]. Though not all strains of *V*. *parahaemolyticus* are pathogenic, *V*. *parahaemolyticus* has several potential virulence factors, such as hemolysin that can lead to cytotoxicity in host cells and severe gastroenteritis if *V*. *parahaemolyticus* is consumed by humans or animals [18–19]. Past studies have shown both minimal [15, 20–21] and significant positive correlations between fecal coliforms and *V*. *parahaemolyticus* as well as other *Vibrio* species, including strains that occur naturally in estuarine environments [14, 22].

Specific toxins primarily associated with pathogenic *V*. *parahaemolyticus* are thermostable direct hemolysin (TDH) and TDH-related hemolysin (TRH) [18–19]. Thermolabile hemolysin (TLH) has also been identified, but is present in both pathogenic and non-pathogenic [11]. As *V*. *parahaemolyticus* has become a serious issue for seafood safety, efforts have been made to identify gene markers associated with pathogenesis of *V*. *parahaemolyticus* and the genes encoding for TDH (*tdh*), TRH (*trh*), and TLH (*tlh*) have been identified [12, 23], allowing for the development of molecular *V*. *parahaemolyticus* detection techniques [24–26]. As *tlh* is found in all known strains of *V*. *parahaemolyticus*, it is considered a presence/absence indicator for *V*. *parahaemolyticus*, whereas *tdh* and *trh* are used to specifically identify pathogenic strains.

*Vibrio parahaemolyticus* has been measured in shellfish in many regions across the US [15–16, 27], but pathogenic *V*. *parahaemolyticus* contamination in shellfish is a relatively recent phenomenon in Northwest Atlantic Ocean waters [12, 15]. Increases in pathogenic *V*. *parahaemolyticus* outbreaks are thought to be due to an invasion of a pathogenic strain indigenous to the Pacific Northwest, as well as elevated water temperatures during summer months in recent years [12]. Post-harvest temperature and handling practices have been shown to largely control levels of pathogenic *V*. *parahaemolyticus* in shellfish, as microbial populations are capable of rapid growth *in vivo* even when exposed to high-temperatures for only a short time [28–29]. While the effect of temperature on *V*. *parahaemolyticus* concentrations has been well described, the role of other environmental factors in controlling pelagic *V*. *parahaemolyticus* concentrations remains limited and such information could be used to develop management plans that ensure food safety [10–11].

To more fully ensure the safety of shellfish, direct measurements of pathogenic *V*. *parahaemolyticus* in contaminated harvest areas are warranted. Data assessing the concentration and distribution of *V*. *parahaemolyticus* in coastal waters could prove to be an important management tool for ensuring shellfish safety as it could provide insight regarding the ecology of *V*. *parahaemolyticus* as well as environmental factors that promote high concentrations. This study addresses this knowledge gap through surveys of total and pathogenic strains of *V*. *parahaemolyticus* in estuaries across the north shore of Long Island, NY, USA, where outbreaks of pathogenic *V*. *parahaemolyticus*-associated with human illnesses occurred in 2014. Beyond quantifying total and pathogenic strains of *V*. *parahaemolyticus*, parallel measurements of multiple indicator bacteria and environmental variables were made. Further, experimental manipulations of temperature and dissolved organic matter were performed to assess their role in growth of total and pathogenic strains of *V*. *parahaemolyticus*. Hypotheses proposed for the study included: 1) total and pathogenic *V*. *parahaemolyticus* concentrations will show no significant correlation with indicator bacteria species and 2) total and pathogenic *V*. *parahaemolyticus* concentrations will be significantly higher after experimental additions of dissolved organic matter and when incubated at temperatures higher than ambient levels.

## Methods

### Field sampling of *Vibrio parahaemolyticus*, environmental parameters, and indicator bacteria

Field samples were collected weekly at four sampling sites located in areas currently uncertified for shellfish harvest within Hempstead Harbor (40.840°N, 73.653°W), Oyster Bay Harbor (40.878°N, 73.528°W), Cold Spring Harbor (40.875°N, 73.471°W), and Northport Harbor (40.890°N, 73.361°W), NY, USA (S1 Fig), as well as limited sampling performed at Huntington Harbor (40.890°N, 73.418°W), NY, USA (S1 Fig), from summer through fall of 2015, the time period when *V*. *parahaemolyticus* contamination and shellfish bed closures had previously occurred in this region [12]. Waterfront areas were accessed from public docks and the collection of small volumes of water is not a regulated activity in New York State. A surface water sample was taken at each site using an autoclave-sterilized, 1-liter polypropylene Nalgene bottle. All samples were immediately transported in coolers and processed within two hours of collection. Dissolved oxygen and temperature levels were recorded *in situ* using HOBO U26-001 dissolved oxygen data loggers and YSI 5920 sondes. Salinity and water temperature were also measured on site using handheld the YSI 556 sonde and turbidity was evaluated using a Secchi disc. *In vivo* fluorescence-based quantification of chlorophyll *a* was determined using a bbe-Moldaenke FluoroProbe II that was calibrated to [30]. Rainfall data was obtained from the National Weather Service monitoring station in Islip, NY, USA, located within 55 km of the sampling locations.

Fecal coliform and enterococci concentrations were measured using US EPA Clean Water Act Section 304(h) approved IDEXX Quanti-tray measurement system, which uses a Most Probable Number (MPN) approach [31]. Using this method, samples are enriched with growth media, separated into sealed Quanti-trays, and incubated overnight. The MPN estimate is then based on the number of positive and negative wells of the Quanti-tray post-incubation [31].

Total and pathogenic *V*. *parahaemolyticus* were quantified using an MPN, multiplex PCR assay that quantified both total and pathogenic strains of *V*. *parahaemolyticus* via distinct genetic markers [13, 21, 26]. The *V*. *parahaemolyticus* MPN procedure adapted from the USFDA Bacteriological Analytical Manual used volumes of 100 ml, 10 ml, and 1 ml in triplicate taken from field samples [32]. Each volume was enriched with 10X alkaline peptone water (10% peptone, pH 8.5), with additions of 11 ml, 1.1 ml, and 0.11 ml of alkaline peptone water to the 100 ml, 10 ml, and 1 ml sample volumes respectively. All volumes were then incubated overnight at 35°C. Following incubation, 200 μl of the enriched samples were transferred to microcentrifuge tubes and placed into a boiling water bath for 10 minutes to lyse any *V*. *parahaemolyticus* cells present [13, 21]. All samples were then immediately stored at -80°C until PCR reactions were performed.

The multiplex PCR procedure used recently published primer sequences for *tlh*, *tdh*, and *trh* designed to aid in distinguishing between *tlh* and *trh* using a standard PCR procedure [26]. Forward and reverse primer sequences were as shown in Table 1. Multiplex primer mixtures were prepared with final concentrations of 1 μM *tlh* and 2 μM of *trh* and *tdh* to aid the amplification of pathogenic markers at low concentrations [24]. A 25 μl PCR mixture was prepared using 12.5 μl GoTaq Green Master Mix, 2.5 μl multiplex primer mixture, 2 μl template DNA, and 8 μl nuclease-free water. PCR temperature cycles began with an initial 3-minute denaturation at 94°C then completed 30 cycles of denaturation at 94°C for 1 minute, primer annealing at 55°C for one minute, and extension at 72°C for one minute. A final extension at 72°C for 5 minutes completed the PCR procedure [24]. All PCR procedures used genomic *V*. *parahaemolyticus* DNA or prior positive field samples as a positive control and nuclease-free water as a negative control. Amplicons were evaluated with gel electrophoresis, using 5 μl of sample on a 2% agarose gel with a current of 85 Watts run for 45 minutes. Total and pathogenic *V*. *parahaemolyticus* were quantified based on the presence or absence of *tlh*, *trh*, and *tdh* at each dilution volume using a customized USFDA MPN procedure [33]. Samples were considered to contain pathogenic strains of *V*. *parahaemolyticus* if either *trh* or *tdh* were present. Samples below detection limits were reported as one-half of minimum detectable levels to aid in visual representation and samples above the detection range were reported at the next most conservative MPN estimate.

**Table 1:** Primer sequences used in the MPN PCR assay.

Gene	Primer sequence	Amplicon size
*Tlh*	F: AGAACTTCATCTTGATGACACTGC	401 bp
	R: GCTACTTTCTAGCATTTTCTCTGC	
*Trh*	F: CATAACAAACATATGCCCATTTCCG	500 bp
	R: TTGGCTTCGATATTTTCAGTATCT	
*Tdh*	F: GTAAAGGTCTCTGACTTTTGGAC	269 bp
	R: TGGAATAGAACCTTCATCTTCACC	

Primer sequences used in the MPN PCR assay for identification of *tlh*, *trh*, and *tdh*.

### Relationships between indicator bacteria, environmental parameters, and *Vibrio parahaemolyticus*

Spearman’s Rank Order regression analyses were performed on data from field samples to identify any correlation between fecal coliform bacteria, enterococci, water temperature, chlorophyll *a*, dissolved oxygen, salinity, turbidity, or rainfall and total or pathogenic *V*. *parahaemolyticus*. Additionally, a logistic regression analysis was used to model the relationship between environmental parameters and the presence or absence of pathogenic *V*. *parahaemolyticus*, with significance determined through a likelihood-ratio test. Confidence intervals for MPN field data were generated through the USFDA MPN procedure [33].

### Experimental incubations

Bottle incubations experiments were performed to understand the influence of temperature and dissolved organic matter (DOM) on the abundance of total and pathogenic strains of *V*. *parahaemolyticus*. To produce DOM for experiments, phytoplankton cultures were utilized. Specifically, 1-liter each of *Chaetoceros muelleri*, *Isochrysis galbana*, *Tetraselmis suecica*, and *Pavlova lutheri* grown in f/2 media (containing nitrogen, phosphorus, silicon, trace metals, B-vitamins, and an iron chelated) at 24°C at 100 μEin m^-2^ s^-1^ at densities of 2 x 10^6^ cells ml^-1^ (save for *Tetraselmis suecica* which had a density of 5 x 10^5^ cells ml^-1^; cells microscopically quantified via a hemocytometer) were centrifuged in 100 ml aliquots for 10 minutes at 10,000 rpm to pellet cells. After removal of the supernatant, the pellets were resuspended in a 50 ml Falcon tube using 10 ml of distilled water and then sonicated (Ultrasonic Power Corporation, Freeport, Illinois. Model 1000L) at 30% power for two one-minute intervals to lyse cells. Microscopic inspection confirmed the lysed of nearly all cells. A second round of centrifugation for 10 minutes at 10,000 rpm at 20°C then took place to pellet any remaining particulate matter, and the supernatant was then filtered through combusted (2 h @ 450°C) Pall, GFF glass fiber filters (0.7 μm) using a syringe to obtain the final DOM for experimentation. The final stock DOM contained 1082, 642, and 427 μM of dissolved organic carbon, dissolved nitrogen, and dissolved phosphorous, respectively. Four, 2-L polycarbonate bottles were filled with water from Cold Spring Harbor and 5 ml of the concentrated dissolved organic matter was added, mimicking the lysis of an algal bloom of ~ 3 x 10^5^ cells ml^-1^, a level previously observed in the sampling region [34–36]. Four separate 2-L bottles containing water from field sites without organic matter additions were used as a control. Bottles were placed in an outdoor, flow-through water bath at the Stony Brook—Southampton Marine Sciences Center, 75 km east of Cold Spring Harbor, which maintained water temperatures and light levels similar to those observed at a 1 m depth in Cold Spring Harbor at the time of sample collection. After 24 h, aliquots were removed to quantify densities of total and pathogenic *V*. *parahaemolyticus* as described above. Two of these experiments were performed on September 15 and 29, 2015.

Temperature experiments were performed using water from Cold Spring Harbor divided into eight 2 L bottles, with four maintained at ambient temperature measured at the field site and four incubated at ~ 3°C above ambient temperature. Temperature was manipulated by using Aqua Logic^®^ In‑Line Heaters in ambient outdoor water baths described above. After 24 h, aliquots were removed to quantify densities of total and pathogenic *V*. *parahaemolyticus* as described for field samples. Two such experiments were performed on October 6 and 20, 2015. Differences in densities of total *V*. *parahaemolyticus* between controls and treatments for both types of experiments were assessed through use of One-Way ANOVAs. Differences in the proportion of samples containing pathogenic *V*. *parahaemolyticus* between treatments were assessed using a two-sample test for equality of proportions. Confidence intervals for experimental results were generated via bootstrapping techniques.

## Results

### Field surveys

Water temperatures followed expected seasonal patterns and were fairly consistent across sampling sites, with Northport Harbor showing slightly higher temperatures in the early portion of the sampling period. Temperatures rose slightly in the first weeks of the sampling period before reaching sustained peak levels in late August, reaching or exceeding 25°C at sampling sites. Thereafter, water temperatures steadily declined into fall, falling below 10°C in the final weeks of sampling in December. Precipitation in the two days prior to sampling was limited during the sampling season. The largest rain event of 0.97 inches occurred in the first week of sampling and rainfall within the two-day window occurred only three additional times over the sampling period (S2 Fig).

**Fig 1:**
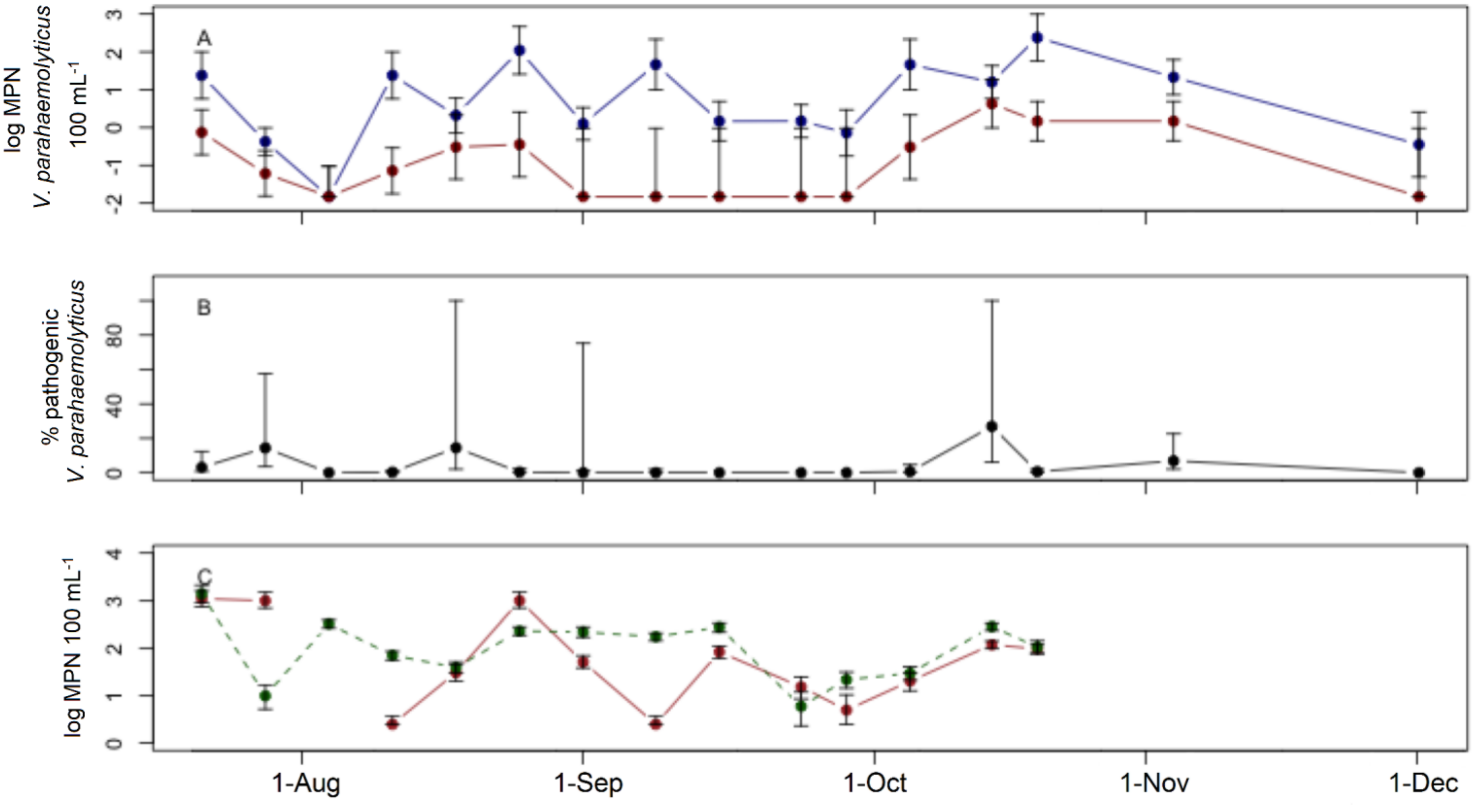
Field survey at Hempstead Harbor. Field survey at Hempstead Harbor. A) Concentrations of total (blue) and pathogenic (red) *V*. *parahaemolyticus*. B) Percent of total *V*. *parahaemolyticus* identified as pathogenic. C) Concentrations of enterococci (solid) and fecal coliforms (dashed). Error bars represent 95% confidence intervals.

Levels of total and pathogenic strains of *V*. *parahaemolyticus* varied in space and time but were generally higher in summer than fall, highest at Cold Spring Harbor, and lowest at Northport Harbor. At Hempstead Harbor, total *V*. *parahaemolyticus* was detectable with week-to-week variation during summer months before a decline in late fall. Total *V*. *parahaemolyticus* ranged from 0.4 to 240 MPN 100 mL^-1^, with a mean concentration of 7.2 MPN 100 mL^-1^ (Fig 1). Pathogenic strains were present in 63% of samples, primarily in the early and latter portions of the sampling period. When detected, pathogenic *V*. *parahaemolyticus* concentrations ranged from 0.1 to 4.3 MPN 100 mL^-1^, with a mean concentration of 0.5 MPN 100 mL^-1^ (Fig 1). The proportion of total *V*. *parahaemolyticus* that was pathogenic ranged from 0 to 28%, and averaged 4.2%. Indicator bacteria concentrations also showed temporal variation over the sampling period with high concentrations in summer months before a decline in the latter portions of the sampling period (Fig 1). Enterococci concentrations ranged from 2.5 to 1100 MPN 100 mL^-1^ with a mean of 51 MPN 100 mL^-1^, while fecal coliform concentrations ranged from 6.0 to 1410 MPN 100 mL^-1^ with a mean concentration of 96 MPN 100 mL^-1^ (Fig 1).

**Fig 2:**
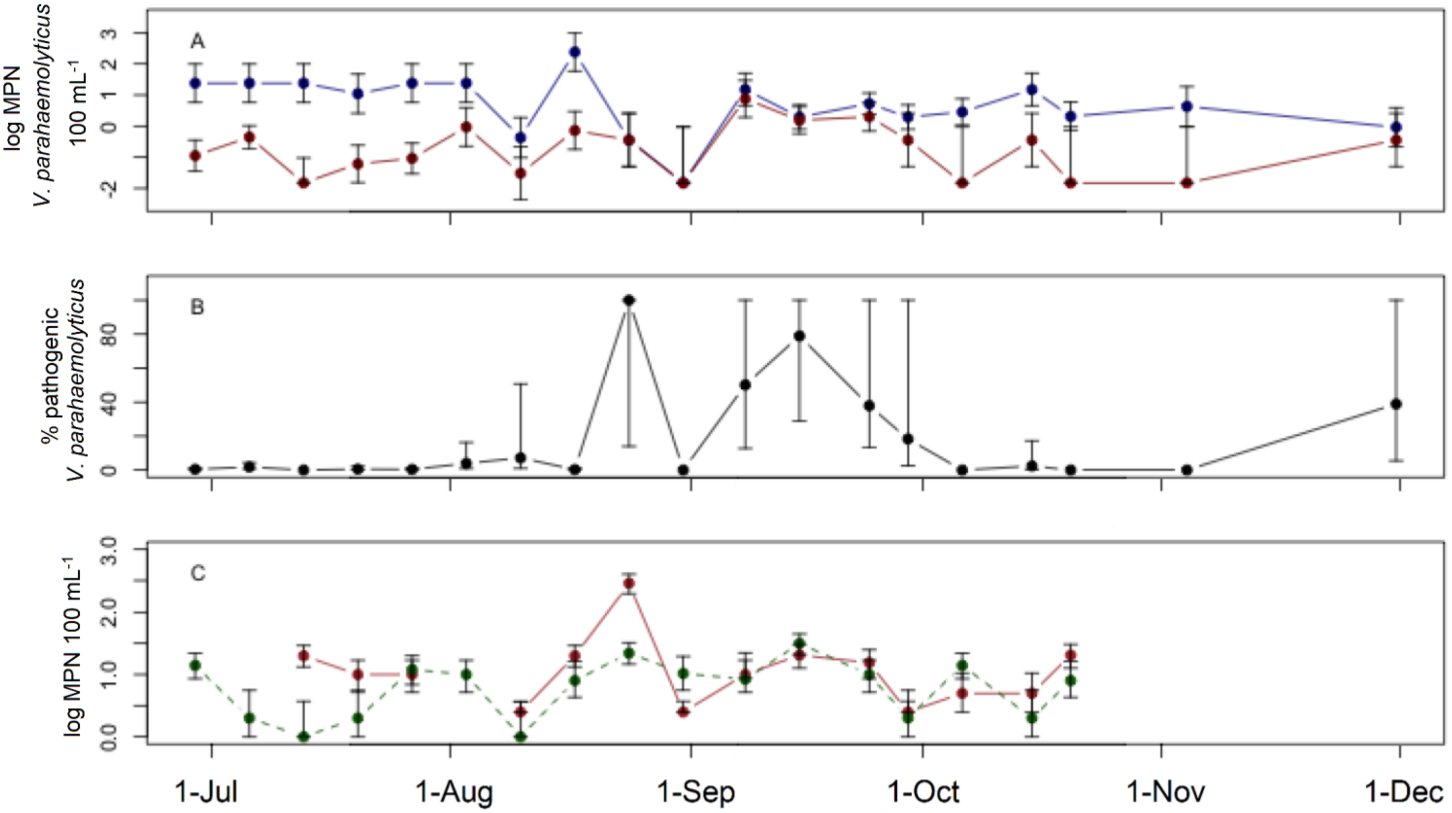
Field survey at Oyster Bay Harbor. Field survey at Oyster Bay Harbor. A) Concentrations of total (blue) and pathogenic (red) *V*. *parahaemolyticus*. B) Percent of total *V*. *parahaemolyticus* identified as pathogenic. C) Concentrations of enterococci (solid) and fecal coliforms (dashed). Error bars represent 95% confidence intervals.

*V*. *parahaemolyticus* was consistently present at Oyster Bay Harbor with the exception of one week late summer. When present, total *V*. *parahaemolyticus* concentrations ranged from 0.4 to 240 MPN 100 mL^-1^, with a mean concentration of 6.5 MPN 100 mL^-1^ (Fig 2). Pathogenic strains were fairly persistent at this site, being found in 74% of samples. When detected, pathogenic *V*. *parahaemolyticus* concentrations ranged from 0.03 to 7.6 MPN 100 mL^-1^ with a mean concentration of 0.4 MPN 100 mL^-1^ (Fig 2). The proportion of total *V*. *parahaemolyticus* that was pathogenic at Oyster Bay Harbor varied from 0 to 100%, though the majority of samples contained only a small proportion of pathogenic strains. Overall, the mean proportion of pathogenic *V*. *parahaemolyticus* was 18%. Indicator bacteria showed more temporal consistency at Oyster Bay Harbor with an expected seasonal trend. Enterococci concentrations ranged from 2.5 to 280 MPN 100 mL^-1^ with a mean concentration of 11 MPN 100 mL^-1^, while fecal coliform concentrations ranged from 1.1 to 32 MPN 100 mL^-1^ with a mean of 5.9 MPN 100 mL^-1^ (Fig 2).

**Fig 3:**
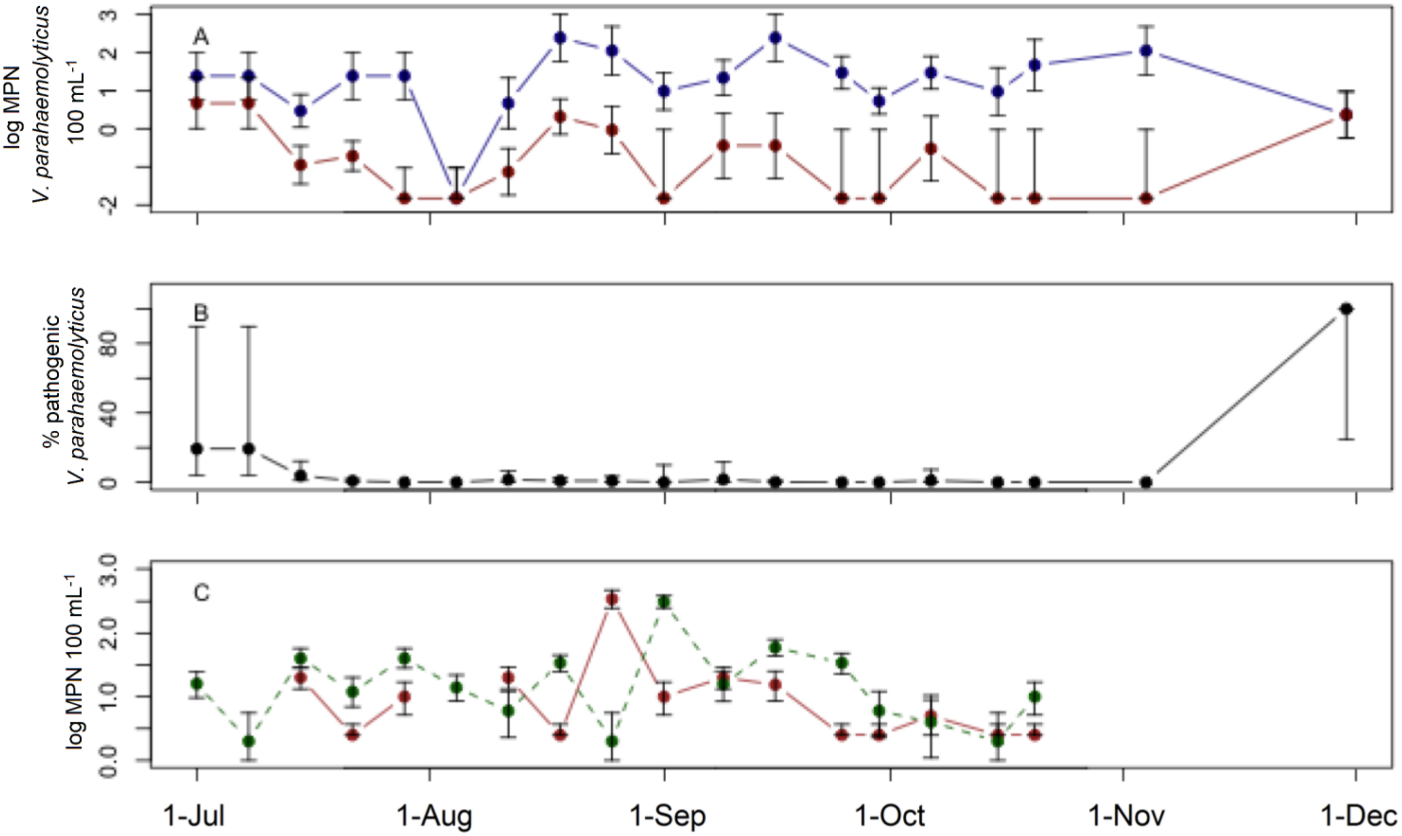
Field survey at Cold Spring Harbor. Field survey at Cold Spring Harbor. A) Concentrations of total (blue) and pathogenic (red) *V*. *parahaemolyticus*. B) Percent of total *V*. *parahaemolyticus* identified as pathogenic. C) Concentrations of enterococci (solid) and fecal coliforms (dashed). Error bars represent 95% confidence intervals.

Cold Spring Harbor had consistently elevated total *V*. *parahaemolyticus* levels, ranging from 2.3 to 240 MPN 100 mL^-1^ with the highest mean concentration among sites of 23 MPN 100 mL^-1^ (Fig 3). Pathogenic *V*. *parahaemolyticus* was found in 58% of samples and, when detected, concentrations ranged from 0.07 to 4.6 MPN 100 mL^-1^ with the highest mean concentrations among sites of 0.6 MPN 100 mL^-1^ (Fig 3). Cold Spring Harbor also showed a broad range in the proportion of total *V*. *parahaemolyticus* comprised of pathogenic strains, ranging from 0 to 100%, with the majority of samples having only a small proportion of pathogenic strains (0 to 10%). The mean proportion of total *V*. *parahaemolyticus* containing pathogenic strains was 7.9%. Indicator bacteria at the site were elevated for much of the sampling period, showing a peak in late summer months before declining in the later portions of the sampling period. Enterococci concentrations ranged from 2.5 to 350 MPN 100 mL^-1^ with a mean concentration of 8.1 MPN 100 mL^-1^, while fecal coliform concentrations ranged from 2.0 to 310 MPN 100 mL^-1^ with a mean concentration of 14 MPN 100 mL^-1^ (Fig 3).

**Fig 4:**
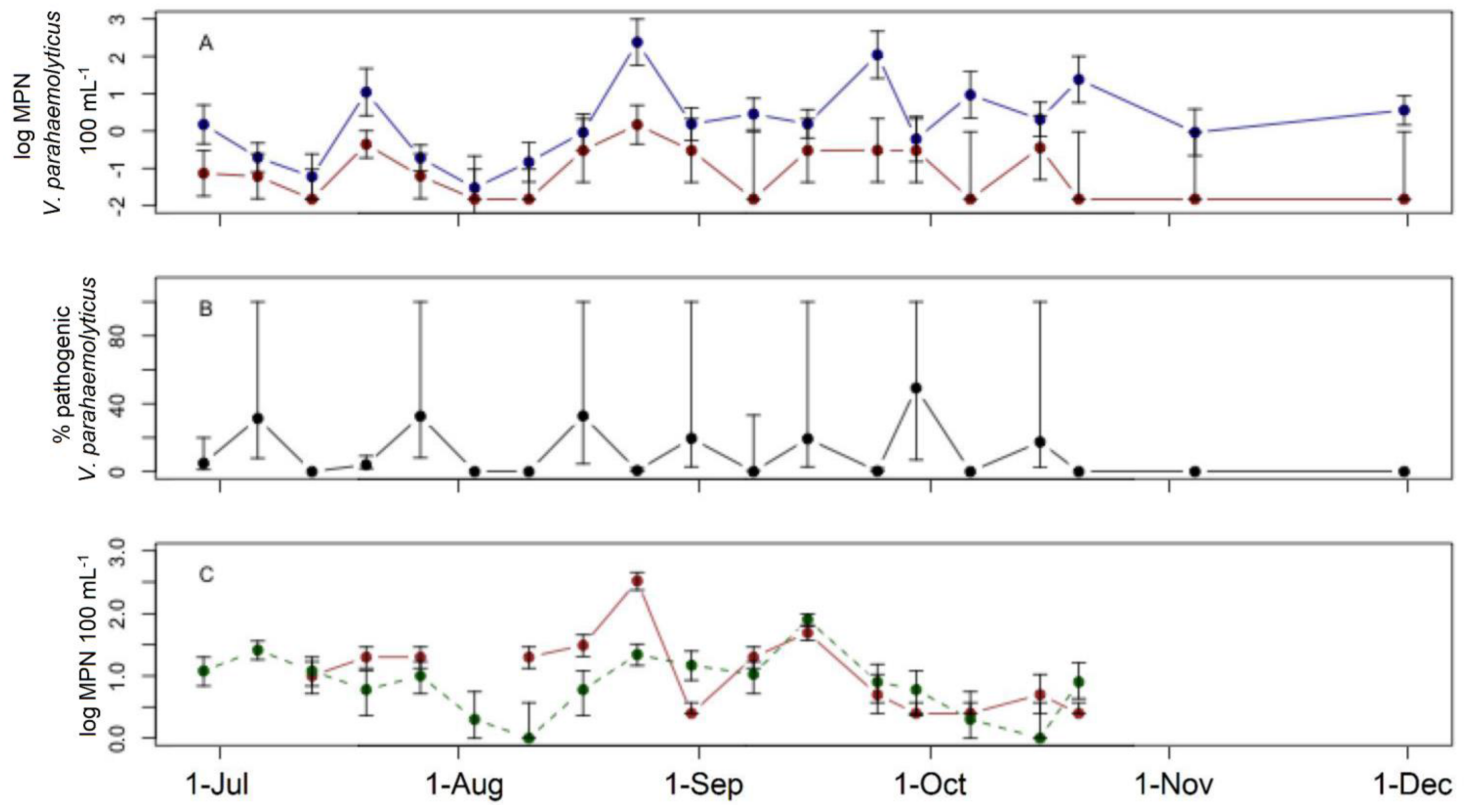
Field survey at Northport Harbor. Field survey at Northport Harbor. A) Concentrations of total (blue) and pathogenic (red) *V*. *parahaemolyticus*. B) Percent of total *V*. *parahaemolyticus* identified as pathogenic. C) Concentrations of enterococci (solid) and fecal coliforms (dashed). Error bars represent 95% confidence intervals.

*V*. *parahaemolyticus* was consistently present in Northport Harbor during late summer and early fall, with lower concentrations seen early in the sampling period. Total *V*. *parahaemolyticus* concentrations ranged from 0.03 to 240 MPN 100 mL^-1^ while the mean concentration was the lowest among the sites at 1.7 MPN 100 mL^-1^ (Fig 4). Pathogenic strains were found in 63% of samples at this site and, when detected, concentrations ranged from 0.06 to 0.7 MPN 100 mL^-1^ with a mean concentration of 0.2 MPN 100 mL^-1^, the lowest average level among the study sites (Fig 4). The proportion of total *V*. *parahaemolyticus* containing pathogenic strains reached a maximum of 49% in September and the mean proportion of total *V*. *parahaemolyticus* that was pathogenic was 11%. Indicator bacteria concentrations in Northport were elevated late summer before declining. Enterococci concentrations ranged from 2.5 to 330 MPN 100 mL^-1^ with a mean concentration of 12 MPN 100 mL^-1^, while fecal coliform bacteria concentrations ranged from 1.0 to 81 MPN 100 mL^-1^ with a mean concentration of 7.4 MPN 100 mL^-1^ (Fig 4).

There were no significant correlations found between total *V*. *parahaemolyticus* and any environmental parameters (Table 2). Pathogenic strains of *V*. *parahaemolyticus*, however, showed significant positive correlations with enterococci densities (*r*(56) = 0.35, *p* <0.01), and rainfall in the two days prior to sampling (*r*(76) = 0.24, *p* < 0.05). Together, these two parameters also produced a significant logistic regression model when applied to pathogenic *V*. *parahaemolyticus* presence-absence data over all sampling sites, *p* <0.01 (Table 3).

**Table 2:** Field survey spearman rank correlations.

	Total *V*. *parahaemolyticus*			Pathogenic *V*. *parahaemolyticus*		
	n	cor	*p*	n	cor	*p*
Enterococci	56	-0.03	0.83	**56**	**0.35**	**<0.01**
Fecal coliforms	68	0.16	0.19	68	0.06	0.6
Water temperature	76	-0.13	0.25	76	0.04	0.72
Rain: Week	76	0.16	0.16	76	0.17	0.15
Rain: Four Day	76	0.05	0.68	76	0.16	0.16
Rain: Two Day	76	0.11	0.35	**76**	**0.24**	**0.04**
Chlorophyll *a*	59	-0.1	0.46	59	-0.2	0.14
Secchi disc depth	36	0.01	0.95	36	0.12	0.5
Salinity	40	0.14	0.39	40	0.08	0.61
Minimum dissolved oxygen	56	0.15	0.97	56	-0.07	0.39

Field survey spearman rank correlations. Significant correlations bolded.

**Table 3:** Logistic regression model.

	Coefficients			
	Estimate	Std. Error	z	*p*
Intercept	-0.091	0.35	-0.261	0.794
Enterococci	0.012	0.009	1.244	0.213
Rain: Two Day	11.43	11.092	1.03	0.303
Null Deviance	74.095 on 55 degrees of freedom			
Residual Deviance	63.297 on 53 degrees of freedom			
Likelihood-ratio	***p* = 0.005**			

Pathogenic *Vibrio parahaemolyticus* presence/absence logistic regression model.

### Experiments

**Fig 5:**
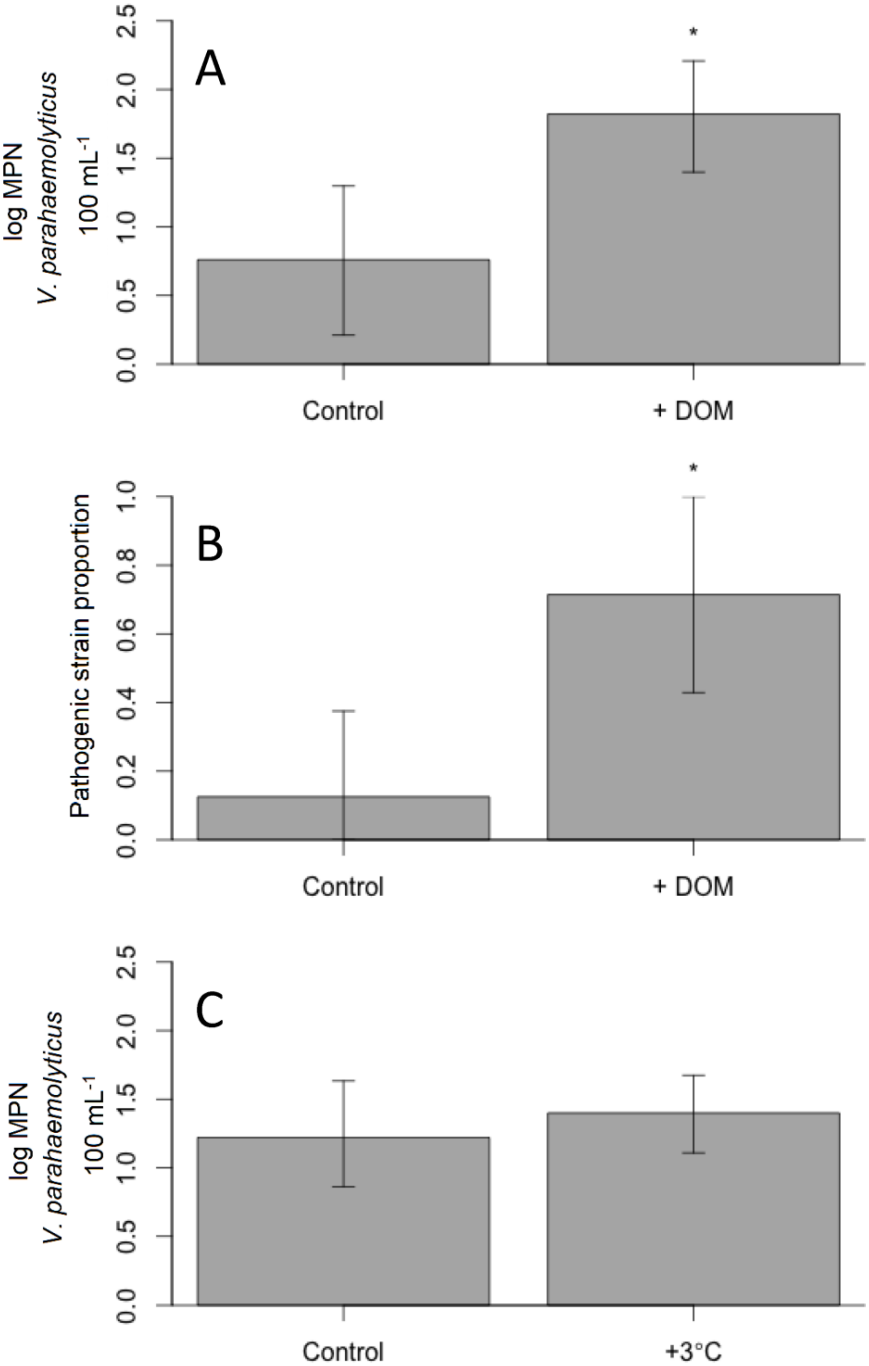
Total *Vibrio parahaemolyticus* concentrations in response to dissolved organic matter and water temperature. A) Concentrations of total *V*. *parahaemolyticus* with and without dissolved organic matter additions; B) Proportion of samples containing pathogenic *V*. *parahaemolyticus* with and without dissolved organic matter additions; C) Concentrations of total *V*. *parahaemolyticus* with and without a water temperature increase. Error bars represent 95% confidence intervals. Significant differences (*p*<0.05) are indicated with an asterisk.

In two experiments, the addition of dissolved organic matter to water samples from Cold Spring Harbor significantly increased total *V*. *parahaemolyticus* concentrations in experimental trials (ANOVA, *F*(1, 12) = 7.65, *p* < 0.02; Fig 5A). Abundance of total *V*. *parahaemolyticus* did not significantly differ between experiments (*F*(1, 12) = 2.27, *p* > 0.05). Dissolved organic matter additions also significantly increased the proportion of samples in experiments containing pathogenic *V*. *parahaemolyticus* (χ^2^ = 5.4018, df = 1, *p* < 0.05; Fig 5B). In contrast, higher temperatures did not significantly alter levels of total *V*. *parahaemolyticus* concentrations (*F*(1, 13) = 0.43, *p* = 0.52) and no differences in abundance were seen between experiments performed (ANOVA, *F*(1, 13) = 0.34, *p* > 0.05; Fig 5C). Pathogenic strains were only seen in one experimental sample from temperature experiments, limiting statistical analyses of these microbes.

## Discussion

*Vibrio parahaemolyticus* is a marine bacterium of growing concern on international, national, and regional levels and yet little is known regarding the ecology or population dynamics of this microbe. During this study, the seasonal dynamics of *V*. *parahaemolyticus* in multiple harbors across the north shore of Long Island were documented and contrasted with indicator bacteria species. Total *V*. *parahaemolyticus* densities were dynamic and statistically unpredictable while the presence of pathogenic *V*. *parahaemolyticus* was significantly correlated with recent rainfall and the indicator microbe, enterococci. Both total *V*. *parahaemolyticus* concentration and the proportion of samples containing detectable levels of pathogenic *V*. *parahaemolyticus* were significantly increased by the addition of dissolved organic matter derived from phytoplankton. Collectively, these findings bring new insight regarding the ecology of total and pathogenic *V*. *parahaemolyticus* in temperate coastal ecosystems.

Indicator bacteria are often measured in coastal ecosystems to protect human health against pathogens. This study afforded the evaluation of efficacy of fecal coliform bacteria and enterococci as indicators of total and pathogenic *V*. *parahaemolyticus*. While total *V*. *parahaemolyticus* concentrations from field samples showed no significant correlation with indicator bacteria, pathogenic *V*. *parahaemolyticus* showed a significant correlation with enterococci. This outcome was somewhat surprising as *V*. *parahaemolyticus* is not an enteric pathogen and therefore is not typically associated with the sewage contamination indicator bacteria are intended to detect [11, 37]. Given that a direct indicator relationship between enterococci and pathogenic *V*. *parahaemolyticus* is unlikely, enterococci may instead be associated with conditions favorable for the growth of pathogenic *V*. *parahaemolyticus*. Given the record of large volumes of wastewater and stormwater that enters western Long Island Sound and surrounding harbors, particularly within the interior of harbors where sampling took place [38–39], it is possible that enterococci serve as a proxy for nutrient and organic matter inputs, the latter of which has been correlated with bacterial production in the region [40]. Importantly, although genetic markers used in this study showed dynamics of pathogenic and non-pathogenic *V*. *parahaemolyticus*, complete genome sequencing of clones could indicate that non-pathogenic *V*. *parahaemolyticus* can carry prophage-like elements, pathogenic islands, or another horizontal gene transfer elements that encode genes related to the virulence. Such observations might could change the perspective between the associations with biotic and abiotic factors.

While significant correlations between total and pathogenic *V*. *parahaemolyticus* and environmental parameters were largely absent, contradicting initial hypotheses, a significant positive correlation was observed between pathogenic *V*. *parahaemolyticus* and rainfall in the two-day period prior to sampling. This result also aligns with experiments showing increased *V*. *parahaemolyticus* concentrations with DOM additions, as short-term increases in rainfall introduce organic matter from surrounding catchments into coastal water bodies [41]. Rainfall has also been shown to increase indicator bacteria concentrations in coastal bays [42–43], and this overlap between rainfall, organic matter input, and levels of indicator bacteria may again explain the observed association of pathogenic *V*. *parahaemolyticus* and enterococci.

During this study, the concentrations and prevalence of total and pathogenic *V*. *parahaemolyticus*, respectively, increased significantly upon the exposure to DOM originating from phytoplankton. DOM is the primary energy source for bacteria in the ocean [44–45] and phytoplankton are the primary source of ocean organic matter [46]. Recent studies of Long Island coastal waters have documented a steady increase in nitrogen levels entering coastal waters and an increasing prevalence of algal blooms [34–35, 47]. Furthermore, many of these algal blooms have been shown to be promoted by the loading of excessive nitrogen from land to sea [34, 36, 48]. Given that total *V*. *parahaemolyticus* concentrations and the proportion of samples containing pathogenic strains of *V*. *parahaemolyticus* increased following the addition of algal organic matter, it would seem that pathogenic *V*. *parahaemolyticus* contamination in shellfish in Long Island coastal waters may be related, at least in part, to rising levels of nitrogen loading that are promoting algal blooms and higher levels of DOM.

The findings of this study have implications for current water quality monitoring strategies and the indicator bacteria paradigm as a whole. The use of fecal coliform bacteria as indicator organisms for monitoring the safety of shellfish growing or harvesting areas relies on the assumption that the bacteria will reliably co-occur with pathogens of concern in the region, and, by extension, that major pathogens are enteric in nature [49]. This study demonstrates that this paradigm does not hold for *V*. *parahaemolyticus*, a non-enteric pathogen responsible for a significant amount of shellfish-related health issues, that showed no association with fecal coliform bacteria concentrations used to ensure food safety. Neither total nor pathogenic *V*. *parahaemolyticus* densities were also not associated with fecal coliform bacteria during this study. This is not necessarily surprising as fecal coliform bacteria were designated as indicator organisms, in part, because they do not reproduce in contaminated environments [9, 50] whereas *V*. *parahaemolyticus* was shown to grow rapidly during incubations with elevated levels of algal organic matter. Pathogenic strains of *V*. *parahaemolyticus* were, however, significantly correlated with enterococci densities, supporting the use of enterococci as indicator bacteria as for shellfish safety, rather than fecal coliforms, a finding consistent with prior studies of bathing beaches [51–52]. However, *V*. *parahaemolyticus* and enterococci have shown no significant relationship in other regions of the United States [16]. Given this uncertainty regarding the presence of different pathogens and their association with common indicator organisms, an alternative approach to water quality monitoring might be one tailored to pathogens present in regional water bodies, especially those that are not enteric in nature, as opposed to broad national standards based on proxies for enteric pathogens alone [9]. Such a strategy for *V*. *parahaemolyticus* monitoring has become more feasible in recent years given the advances in molecular detection techniques, exemplified by the assay presented within this study that employs a standard MPN-PCR procedure. Performing these more targeted assays in parallel with current indicator organism monitoring would provide more robust standards of shellfish safety with regard to non-enteric pathogens such as *V*. *parahaemolyticus* that can pose equal or greater risks to human health than most traditionally monitored enteric pathogens, and examples of such strategies have been implemented in a number of shellfish harvest regions already.

Traditionally, concentrations of bacterial human pathogens in coastal waters parallels water temperatures [27, 53–54]. During this study, a clear temperature-dependent pattern in concentrations of total or pathogenic *V*. *parahaemolyticus* was not apparent until perhaps late fall when multiple sampling sites showed a sharp decline in total and pathogenic *V*. *parahaemolyticus* densities. This pattern is suggestive of a minimal threshold temperature effect on *V*. *parahaemolyticus* abundances rather than a simple linear relationship with temperature, and past studies have shown minimal detection below 15°C [10, 55]. The relative uncertainty surrounding pathogenic *V*. *parahaemolyticus* concentrations as well as the potential for rapid growth of *V*. *parahaemolyticus* when encountering excessive DOM pose challenges for future management of shellfish growing areas affected by this microbe. It is important to note that the majority of pathogenic *V*. *parahaemolyticus* growth in shellfish occurs post-harvest as the bacteria rapidly multiply *in vivo* if shellfish are exposed to warmer temperatures [28]. Thus, control measures designed to reduce the risk of pathogenic *V*. *parahaemolyticus* buildup have focused on shellfish handling, including shading and refrigeration of shellfish once harvested [11, 55]. Given the complexities in growth dynamics of *V*. *parahaemolyticus* strains, if such temperature control measures are employed effectively it is possible that future monitoring of *V*. *parahaemolyticus* could be based around periodic presence/absence sampling, based on either limits of detection or threshold concentrations, to identify at-risk areas in which shellfish handling should be more closely controlled. The MPN assay presented here and in other studies could be employed in such a sampling regime; it is likely that such methods will continue to improve as molecular techniques continue to be refined. For example, more sensitive molecular techniques such as qPCR have also enabled analysis of the ecology of total and pathogenic *V*. *parahaemolyticus* [24–26], knowledge of which will prove critical in refining future management strategies. The emergence of digital PCR as a tool to track pathogenic microbes may also prove to be a useful management tool [56]. Quantitative models such as the one presented within this study, showing a significant relationship between enterococci concentrations, rainfall, and the presence of pathogenic *V*. *parahaemolyticus*, provide insight into potential environmental drivers of pathogenic *V*. *parahaemolyticus*. Future studies should aim to further refine such models to provide a predictive framework for management efforts.

The observed increase in total and pathogenic *V*. *parahaemolyticus* in the presence of elevated DOM levels and the correlation of pathogenic strains with rainfall also highlights the need for holistic coastal management practices in reducing pathogen risk. Inputs of organic matter to coastal water bodies have been associated with detrimental ecological phenomena including hypoxia and harmful algal blooms [57–59] and results within this study indicate that organic matter may also contribute to the proliferation of marine pathogens, further impacting coastal resources. Similarly, it has long been known that runoff within coastal environments can enhance levels of multiple classes of pathogens [5] as well as deliver high levels of nutrients that promote algal blooms [57, 60] and, in turn, enhance DOM levels [46, 57]. Hence, coastal management efforts that seek to restrict excessive nutrient loading from run-off and other sources aimed at restricting algal blooms and hypoxia seem likely to also minimize abundance of *V*. *parahaemolyticus*.

## Supporting information

S1 Fig Sample sites. Sampling sites used in the *Vibrio parahaemolyticus* survey, located at Hempstead Harbor (HMP), Oyster Bay Harbor (OBH), Cold Spring Harbor (CSH), Huntington Harbor (HNT), and Northport Harbor (NPH).
(TIFF)

S2 Fig Water temperatures at sampling sites. Water temperature recorded at individual sampling sites and precipitation accumulated in the two days prior to sampling recorded by the Islip, NY, National Weather Service station over the sampling period.
(TIFF)
