# Supplementary figures and images for: The ability of algal organic matter and surface runoff to promote the abundance of pathogenic and non-pathogenic strains of Vibrio parahaemolyticus in Long Island Sound, USA
Source: PLoS One. 2017 Oct 11;12(10):e0185994. doi: 10.1371/journal.pone.0185994 (PMC5636122; doi:10.1371/journal.pone.0185994)

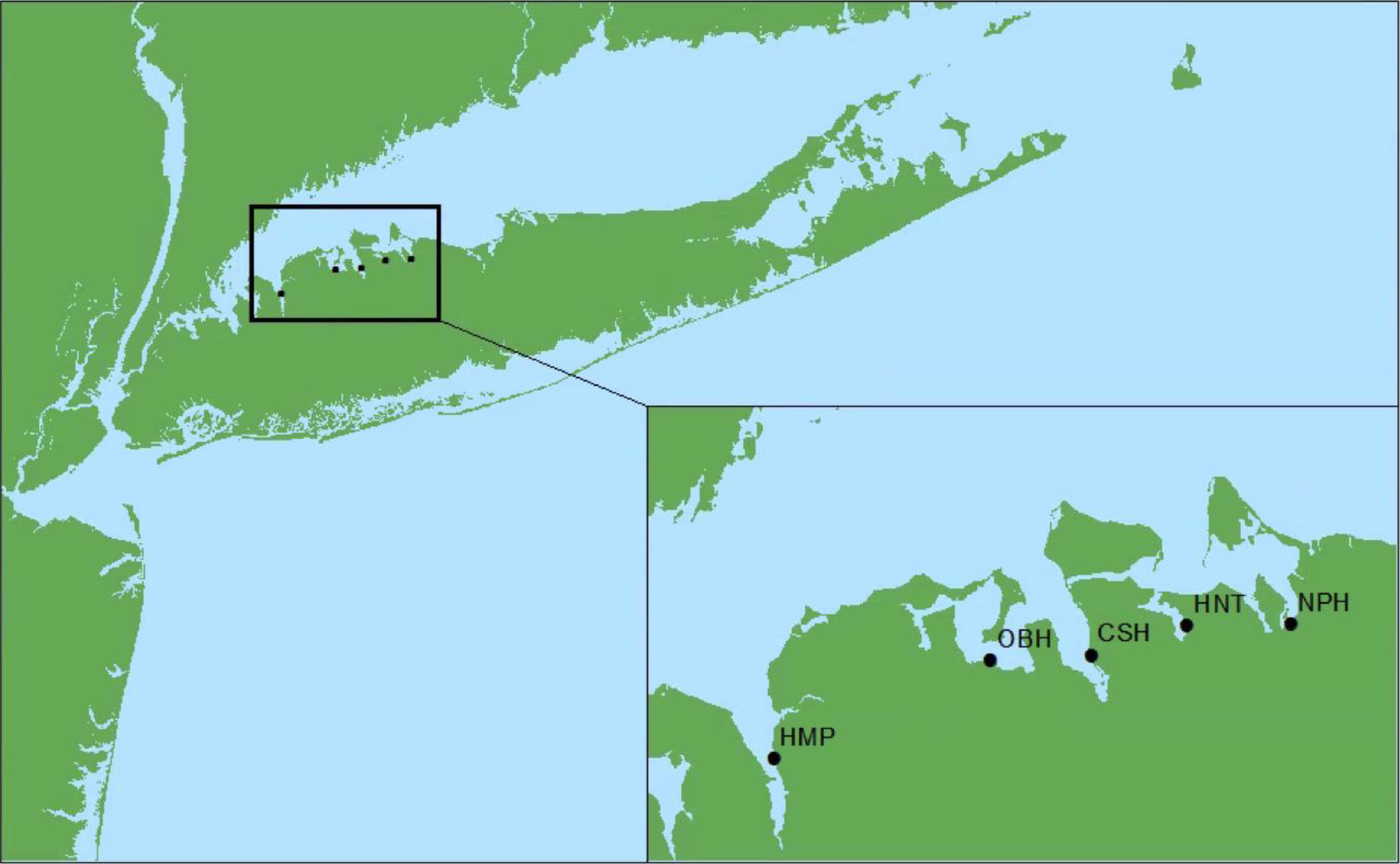

Supplement: S1 Fig — Sampling sites used in the Vibrio parahaemolyticus survey, located at Hempstead Harbor (HMP), Oyster Bay Harbor (OBH), Cold Spring Harbor (CSH), Huntington Harbor (HNT), and Northport Harbor (NPH). (TIFF) [file pone.0185994.s001.tiff]

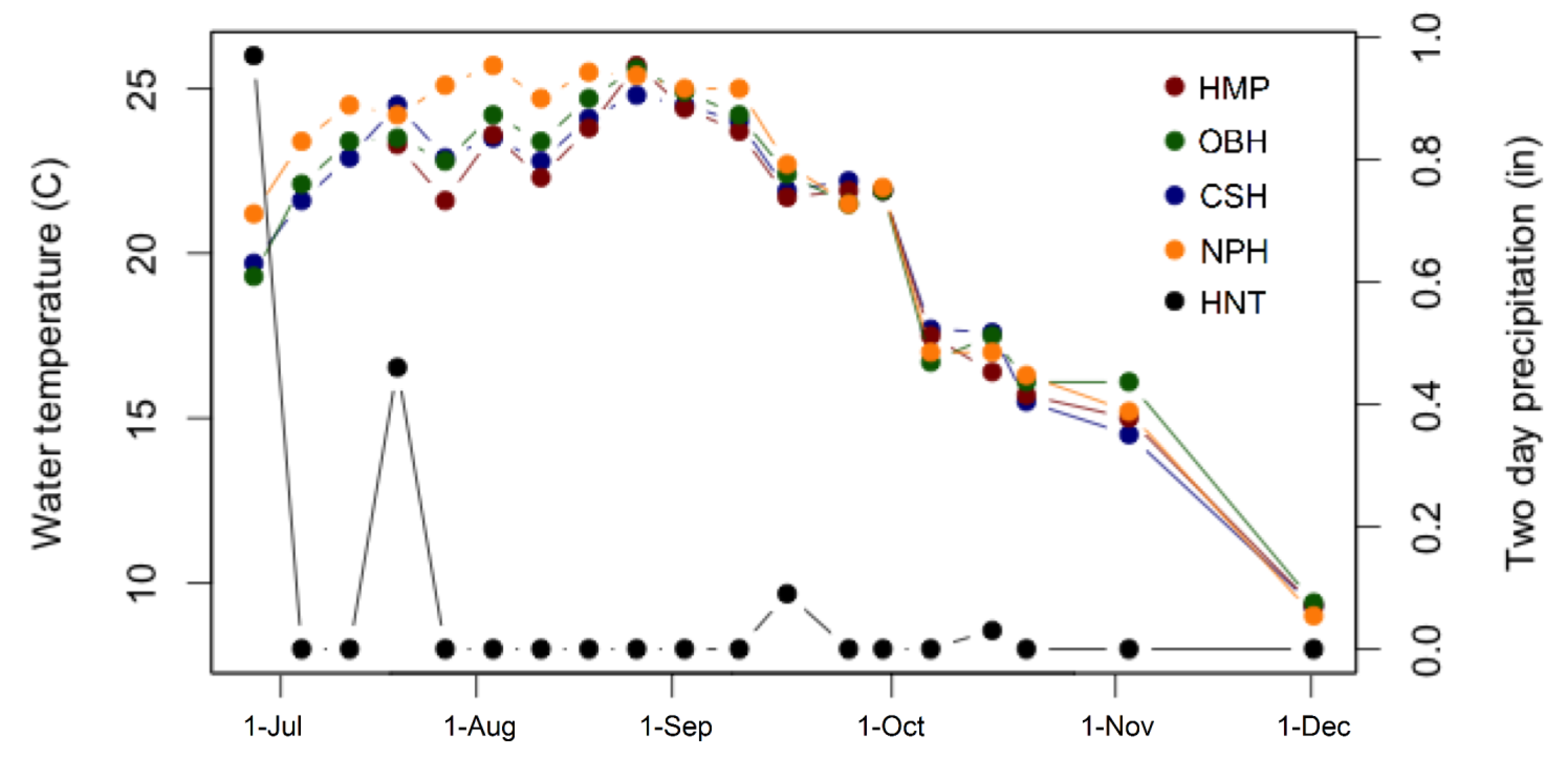

Supplement: S2 Fig — Water temperature recorded at individual sampling sites and precipitation accumulated in the two days prior to sampling recorded by the Islip, NY, National Weather Service station over the sampling period. (TIFF) [file pone.0185994.s002.tiff]
